# NanoThermoMechanical AND and OR Logic Gates

**DOI:** 10.1038/s41598-020-59181-2

**Published:** 2020-02-12

**Authors:** Ahmed Hamed, Sidy Ndao

**Affiliations:** University of Nebraska - Lincoln, Mechanical and Materials Engineering Department, Lincoln, Nebraska 68588-0526 USA

**Keywords:** Information theory and computation, Mechanical engineering

## Abstract

Today’s electronics cannot perform in harsh environments (e.g., elevated temperatures and ionizing radiation environments) found in many engineering applications. Based on the coupling between near-field thermal radiation and MEMS thermal actuation, we presented the design and modeling of NanoThermoMechanical AND, OR, and NOT logic gates as an alternative, and showed their ability to be combined into a full thermal adder to perform complex operations. In this work, we introduce the fabrication and characterization of the first ever documented Thermal AND and OR logic gates. The results show thermal logic operations can be achieved successfully through demonstrated and easy-to-manufacture NanoThermoMechanical logic gates.

## Introduction

Today’s electronics have limited performance and reliability in harsh environments (e.g., elevated temperatures and ionizing radiation environments) found in many engineering applications such as space exploration (e.g., Venus) and geothermal energy exploitation deep beneath the earth; consequently, developing alternative computing technologies is necessary. Thermal computing, data processing based on heat instead of electricity, is proposed as a practical solution and opens a new scientific area at the interface between thermal and computational sciences. The traditional linear and passive thermal components, such as thermal resistors and capacitors, are not sufficient to introduce an integrated thermal logic circuit. It is needed to realize switchable and nonlinear thermal components as their electronic counterparts, which leads to tunable thermal control devices and paves the way for thermal computation technology and thermal information treatment.

Much research efforts have been done to realize thermal diodes, switches, transistors, and thermal logic gates^1–3^. The non-linear behavior of the temperature/phase-dependent thermal conductivity of certain materials was successfully employed to demonstrate thermal switch and regulators^4–9^. Additionally, thermal switches and regulators were realized by tailoring heat conduction through solid/solid and solid/liquid physical contact^10,11^, and by manipulating convection heat transfer mechanisms^12–15^. Another research efforts, which employed thermal radiation, were promising solutions^16–21^. However, the challenge is to develop individual thermal rectifiers or diodes and thermal logic circuits that are not limited to a small operating temperatures or specific materials. Previously, we built and simulated a thermal calculator based on clustered NanoThermoMechanical logic gates that could perform similar operations as their electronic counterparts. We presented the design and modeling of the NanoThermoMechanical AND, OR, and NOT logic gates, achieved through the coupling between near-field thermal radiation (NFTR) and MEMS thermal actuation^22^. NFTR transfers heat via thermal radiation between two surfaces separated by a very small vacuum gap (i.e., comparable to the radiation wavelength). NFTR’s intensity increases exponentially with a decreasing separation gap. Based on this design, we present here the fabrication and characterization of the NanoThermoMechanical AND and OR logic gates.

## Design and Methodology

**Figure 1:**
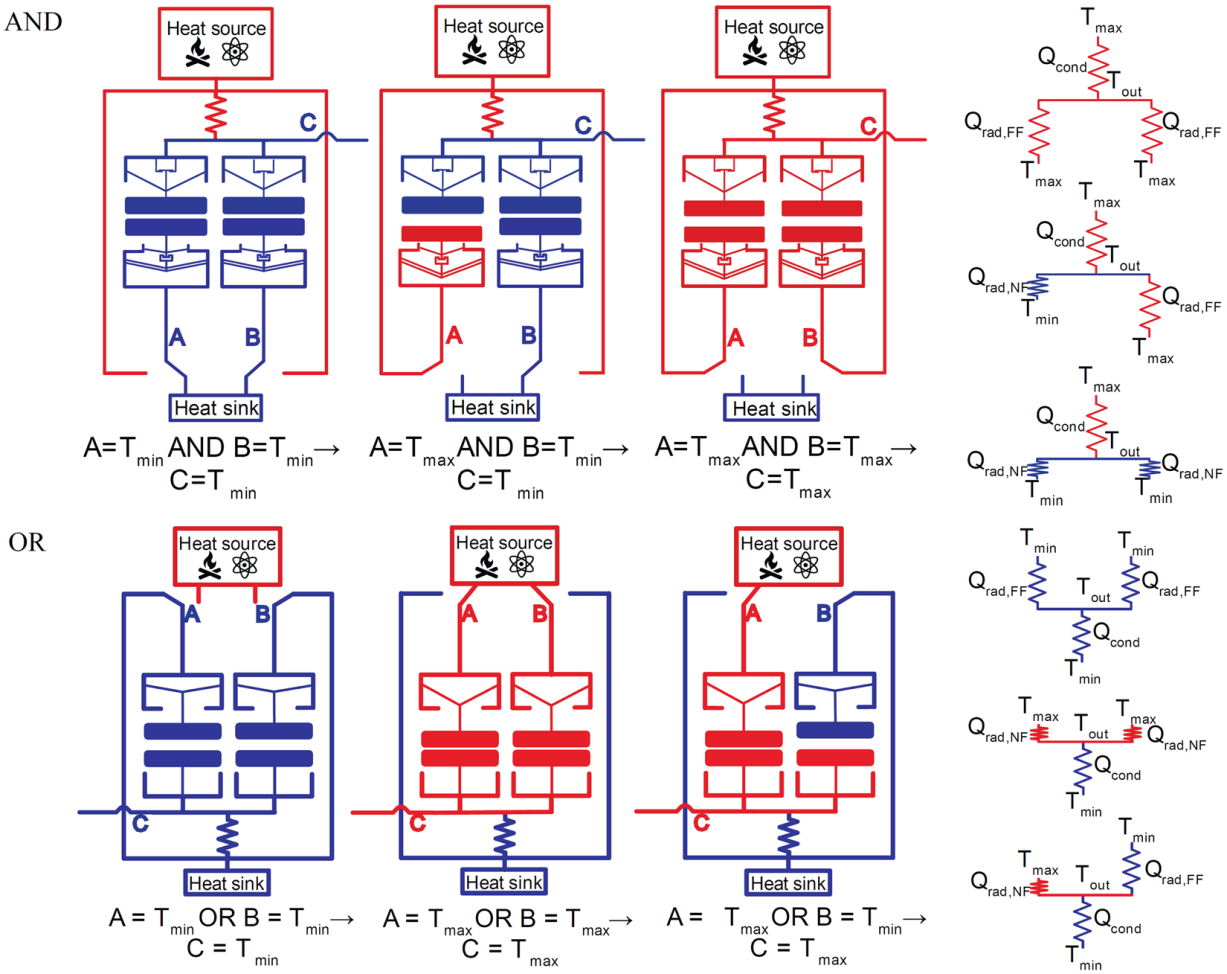
Schematic drawings of the thermal AND and OR logic gates with the heat transfer circuits.

Based on the concept of coupling NFTR and thermal actuation of a chevron beam actuator, thermal AND and OR gates are constructed using a combination of two thermal diodes and a fixed-value conduction thermal resistance (i.e., solid beams with tailored thermal conductance)^22^. Step-by-step operation of the AND and OR thermal gates can be found on Fig. 1. For the AND gate, the upper terminals (output) are connected together to a fixed conductive resistance, which is connected to the heat source. Consequently, the temperature of the output terminal C is a result of the heat balance between the inward conduction heat flow from the heat source $({Q}_{cond})$ and the outward radiation heat flows $({Q}_{rad,NF}\,or\,{Q}_{rad,FF})$ to the lower input terminals (A and B). To achieve the required functionality of the AND gate, output upper terminal C needs to be at its bottom position when ${T}_{A}={T}_{min}\,or\,{T}_{B}={T}_{min}$, regardless the temperature of terminal C, to achieve the minimum separation distance between terminals (i.e., near-field thermal radiation). In addition, terminals A and B need to be separated by large enough gap from terminal C (i.e., far-field thermal radiation) when ${T}_{A}={T}_{max}\,or\,{T}_{B}={T}_{max}$, regardless the temperature of terminal C. In other words, nonlinearity in the terminals’ thermal displacement is required. The upper terminal (output) of the thermal AND gate must feature a reduced $(\frac{\beta }{\alpha } < 1)$ thermal expansion while the lower terminals (inputs) must experience amplification $(\frac{\beta }{\alpha } > 1)$, where *α* is the displacement rate of the terminal between ${T}_{room}$ and ${T}_{min}$, and *β* is the displacement rate of the terminal between ${T}_{min}$ and ${T}_{max}$.

The reducing mechanism can be achieved by spring-assisted chevron beams. The terminal surface is connected to the chevron beams and separated by a small gap from a spring-loaded stopper. Through initial heating, the terminal surface is displaced downwards with a certain expansion rate *α* due to the thermal expansion of the chevron beams. Further heating to a certain designed temperature, the chevron comes in contact with the spring-loaded structure which reduces the expansion rate of the terminal surface to *β* $(\beta  < \alpha )$ proportional to the spring constant and effectively achieving the desired reducing mechanism. As for the amplification mechanism, it can be achieved via two interlocked cascading chevrons with different arm lengths and separated by a small gap. The terminal surface is connected to the short arms chevron. Through heating, the terminal surface is displaced downwards with a certain expansion rate *α*, smaller than the expansion rate of the long arms chevron. Further heating to a certain designed temperature causes the two chevrons to interlock and for the terminal surface to expand at a higher rate *β*, $(\beta  > \alpha )$, effectively achieving the desired amplification mechanism.

For the OR gate, the lower terminals (output) are connected together to the heat sink through a fixed conductive resistance^22^. Consequently, the temperature of the output terminal C is a result of the heat balance between the inward heat flows $({Q}_{rad,NF}\,or\,{Q}_{rad,FF})$ from the lower input terminals (A and B) and the outward heat flow to the heat sink $({Q}_{cond})$. The temperature of each of the input terminals can be controlled independently by choosing to connect the terminals to either the heat source $({T}_{max})$ or the heat sink $({T}_{min})$.

### Microfabrication process

**Figure 2:**
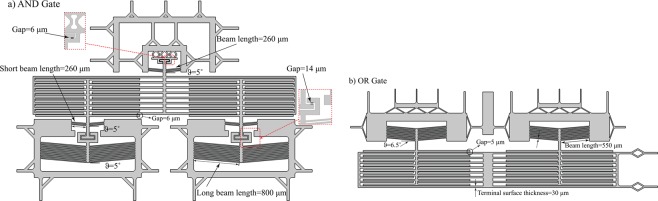
Schematic of the proposed NanoThermoMechanical (**a**) AND and (**b**) OR logic gates.

**Figure 3:**
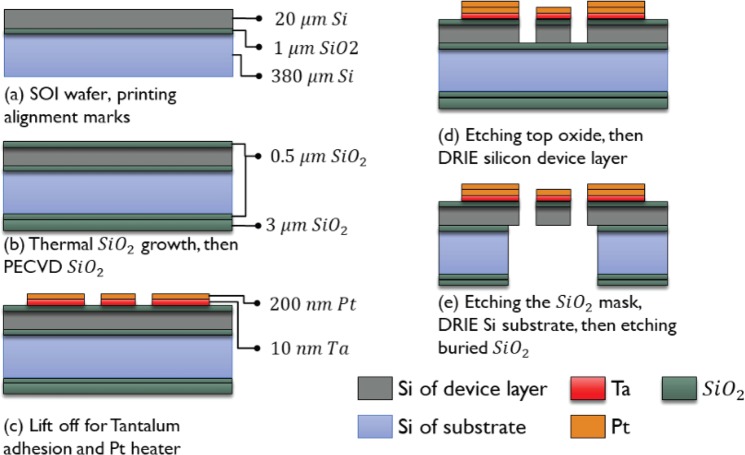
Microfabrication steps of the NanoThermoMechanical AND and OR logic gates.

The proposed microdevices were fabricated using cleanroom standard microfabrication techniques starting with a four-inch-diameter <100> silicon on insulator (SOI) wafer^16^. The SOI wafer consisted of a 400-μm thick handle silicon substrate, a 1-μm thick buried silicon dioxide layer, and a 20-μm thick boron-doped silicon device layer. Figure 2 presents schematic of the proposed microdevices including fabricated dimensions. Figure 3 shows the steps of the process flow adopted for the microfabrication of the NanoThermoMechanical logic gates. First step, after a cleaning of the wafers, a 0.5-μm thick silicon dioxide film (acting as an electrical insulator) was thermally grown by wet oxidation in a furnace at 1100 C° (Fig. 3b) on both sides of the wafer. Then an additional 3-μm thick film of silicon dioxide was needed on the substrate’s backside to serve as an etching mask during the backside etching step. This additional oxide film was deposited via plasma-enhanced chemical vapor deposition (PECVD). The microheaters (200-nm thick platinum and 10-nm thick tantalum as adhesion layer) were patterned on top of the device layer using lift-off photoresist and E-beam evaporation as shown in Fig. 3c. To form the suspended microstructures (Fig. 3d), the 0.5-μm thick thermal silicon dioxide layer was removed through reactive ion etching and the silicon device layer is removed through deep reactive ion etching. To release the final suspended structures, backside etching was performed first on the silicon dioxide, the silicon handle wafer, then the buried silicon oxide (Fig. 3e).

**Figure 4:**
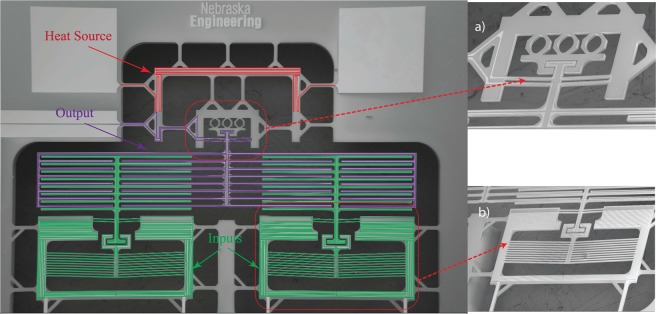
SEM images of the micro-structured thermal logic AND gate including: (**a**) the reducing and (**b**) the amplification mechanism.

**Figure 5:**
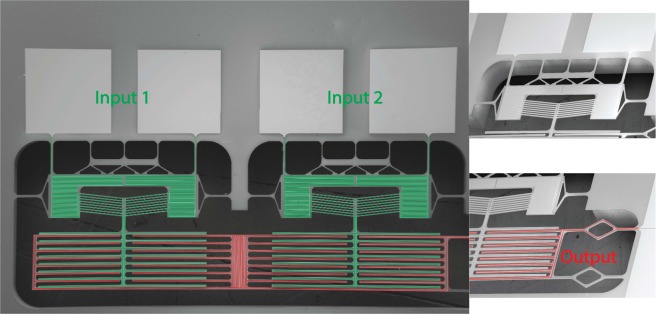
SEM images of the micro-structured thermal logic OR gate including two inputs (chevron beams) and output (fixed terminals).

We designed three photolithography masks: platinum microheaters, silicon front side microstructures, and silicon backside etching. These masks were employed through the microfabrication process flow adopted to fabricate the designed thermal gates. Figures 4 and 5 show the successful microfabrication of the thermal AND and OR gates, respectively, including the reducing and the amplification mechanisms for the thermal AND gate.

## Experimental Procedure and Measurements

The characterization and heat transfer measurements of the thermal logic gates were performed inside a vacuum probe station at vacuum levels below 10^−5^ mbar, in order to eliminate convection and conduction heat losses^16^. The platinum microheaters patterned on the mechanisms were powered independently via two source-meter units (Keithley 2602 B and Keithley 2611 B). A careful temperature coefficient of resistance (TCR) calibration was used to determine the microstructures’ temperatures from knowing the electrical resistance of the microheaters. To assure the stability of the TCR relationship, we annealed the microheaters many times by setting the chuck temperature to 750 K and supplying the maximum allowable current to the microheaters. The TCR calibration was carried out by varying the temperature of the chuck (which holds the microdevice inside the vacuum chamber) from room temperature to 750 K and measuring the corresponding microheaters’ electrical resistances. Then, the resistance of each microheater was fitted to the corresponding temperature using a quadratic relationship^23^.

**Figure 6:**
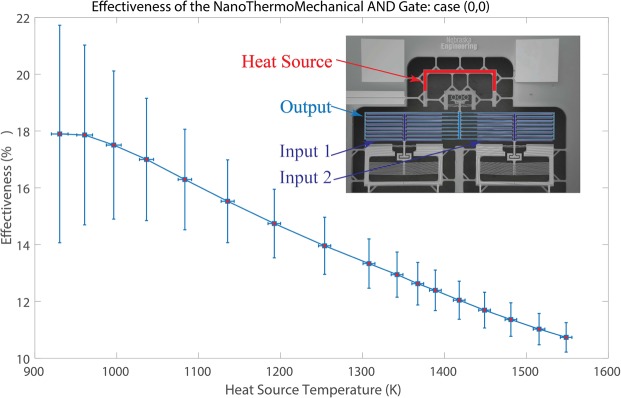
The effectiveness of the NanoThermoMechanical AND gate over the range of the heat source temperature for the case (0,0).

**Figure 7:**
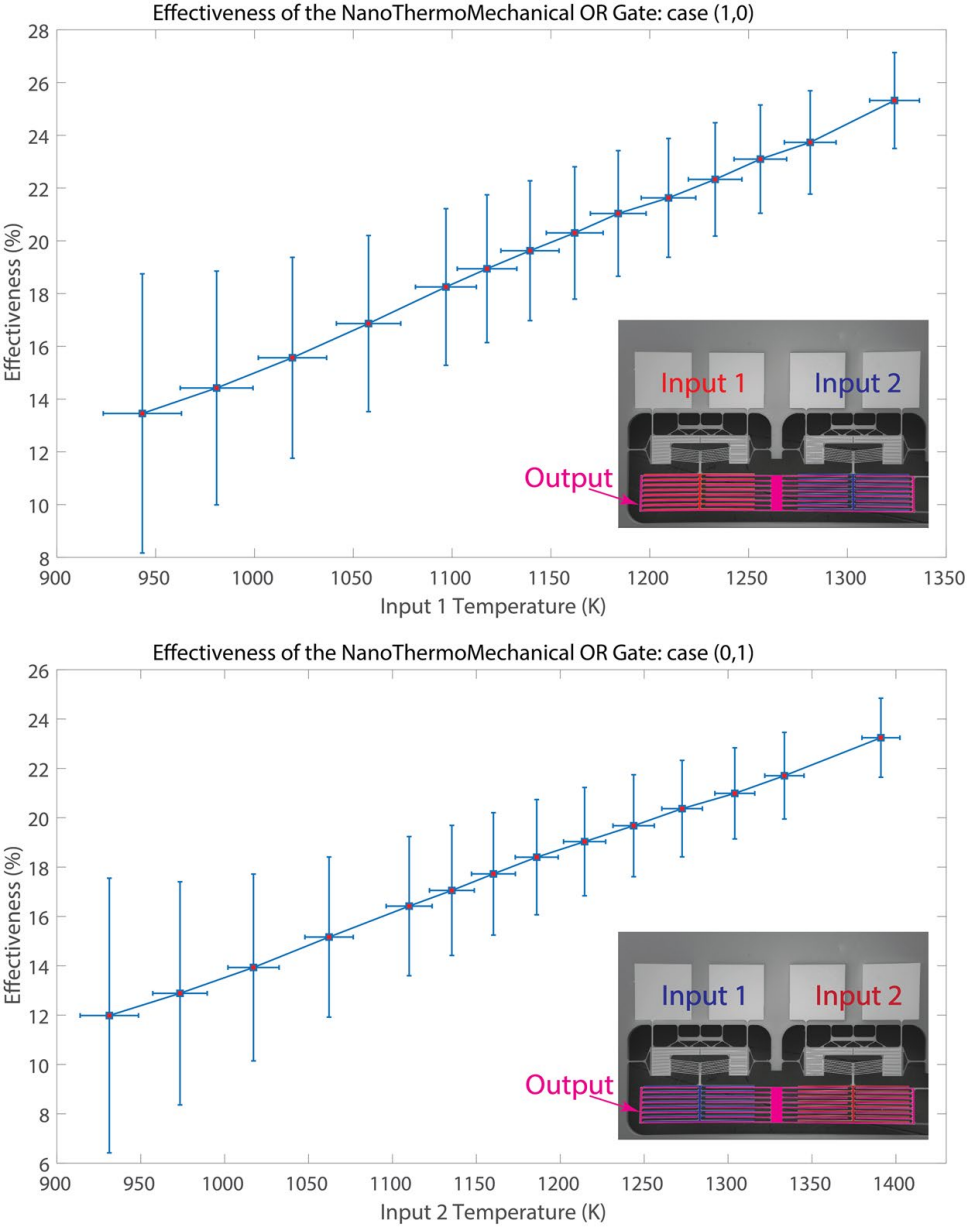
The motion evolution of the non-linear expansion mechanisms over the range of the operating temperatures: (**a**) the spring-assisted reduction and (**b**) the cascading chevrons amplification mechanisms.

Throughout the experiments, the electrical current was supplied gradually through the microheaters over the mechanisms by a step of 0.1 or 0.25 *mA*. The voltage, resistance, and dissipated power of the microheaters were measured at each step of the supplied current. According to the technique published by Moffat^24^ and based on the datasheet documents of the source-meters^25,26^, the uncertainties in the voltage, current, resistance, and dissipated power were estimated to be in the ranges of 0.05–0.06 *V*, 0.6–0.7 *μA*, 165–350 Ω, and 0.1–0.6 *mW*, respectively. Due to the high resistances of the heaters, the experiments were performed at high chuck temperatures, which helped in reducing the required power to actuate the mechanisms. Moreover, our vacuum probe station includes four probes, so just two heaters could be characterized simultaneously. Consequently, for the thermal AND logic gate, we could just present thermal results for the case when the two inputs were at ${T}_{min}$ (i.e., 0,0 case), since two probes were used for supplying the heat source heater and the other two probes were used for measuring the output heater. However, videos of the mechanism for (0,1) and (1,0) cases are included in the supplementary material. Additionally, there are videos that illustrate the non-linearity of the output and input terminals. As shown in Fig. 6, the effectiveness is represented as a function of the heat source temperature. We define the effectiveness, ${\epsilon }$, of the thermal logic gates as ${\epsilon }=\frac{{T}_{out}-\,{T}_{min}}{{T}_{max}-\,{T}_{min}}$, where ${T}_{out}$ is the output terminals temperature, ${T}_{min}$ is the minimum operating temperature (i.e., the chuck temperature) and ${T}_{max}$ is the maximum operating temperature (i.e., the heat source temperature). It can be illustrated that the higher the heat source temperature, the lower the effectiveness that can be achieved. The effectiveness decreased from 17.9% to 10.7% by increasing the heat source temperature from 930 K to 1549 K. This is because at a higher heat source temperature, the output terminals get closer to the input terminals and near field radiation effects become important. Figure 7 presents the motion evolution of the non-linear expansion mechanisms ((a) the spring-assisted reduction and (b) the cascading chevrons amplification) employed in the NanoThermoMechanicl AND logic gate with increasing microstructure temperature.

**Figure 8:**
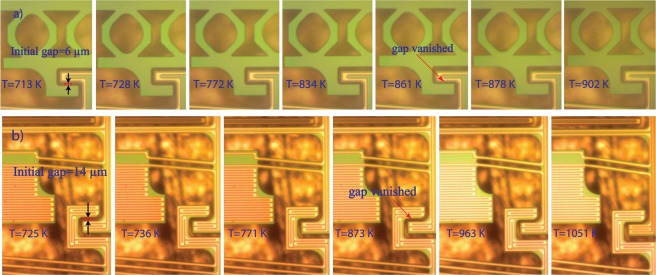
The effectiveness of the NanoThermoMechanical OR gate over the range of input temperatures for: (**a**) case (1,0) and (**b**) case (0,1).

For the thermal OR logic gate, two probes were used for heating one of the two inputs, and the other two probes were used for measuring the temperature of the output heater. Consequently, temperature results for the cases of (1,0) and (0,1) could be presented for the thermal OR logic gate. However, video of the mechanism for (0,1) case is included in the supplementary material. The effectiveness of the gate for these two cases is shown in Fig. 8a,b as a function of the input temperature. It can be illustrated that the higher the input temperature, the higher the effectiveness that can be achieved. For the (1,0) case, the effectiveness increased from 13.5% to 25.3% with increasing input temperature from 943 K to 1324 K. For the (0,1) case, the effectiveness increased from 12.0% to 23.2% with increasing input temperature from 931 K to 1391 K. This is because at a higher input temperature, the input terminals get closer to the output terminals, making near-field radiation the dominant heat transfer mechanism.

**Figure 9:**
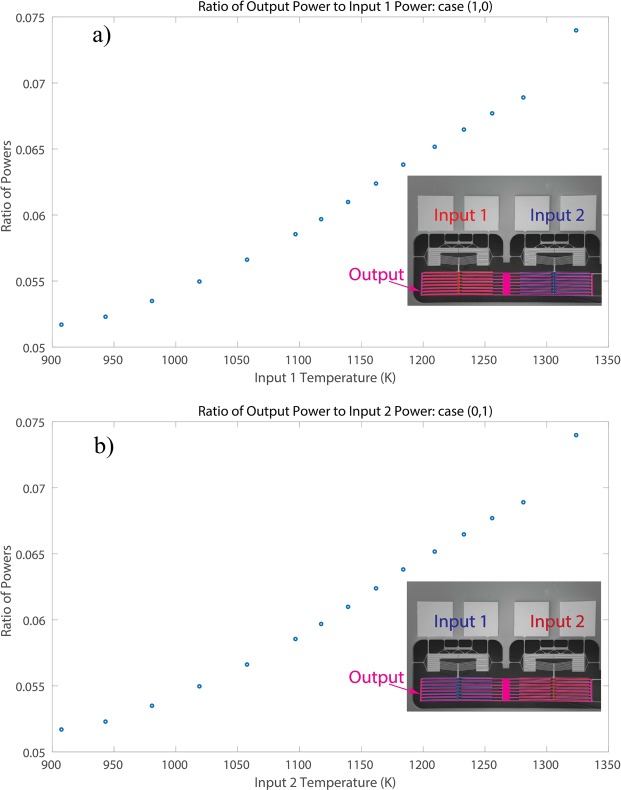
The ratio of the output net power to the input power of the NanoThermoMechanical OR gate over the range of input temperatures for: (**a**) case (1,0) and (**b**) case (0,1).

The ratio between ${Q}_{out}$ (the net power transferred to the output terminals) and ${Q}_{in}$ (the supplied power to the input terminals) is shown in Fig. 9a,b. It is illustrated that by increasing the input temperature, the ratio of the powers increases because of the near-field radiative heat transfer. This ratio can be enhanced by reducing the conduction losses through the microdevice supports and the radiation losses to the chamber. It is worth mentioning that by conducting the experiment of the (1,1) case, where the two inputs are powered to high temperature, the effectiveness is expected to reach higher values.

## Conclusions

In this paper, we presented the design, microfabrication and characterization of first ever documented thermal AND and OR logic gates. The desired non-linearities of associated NanoThermoMecahnical mechanisms were achieved using novel and easy to fabricate chevron mechanisms consisting of spring-assisted reduction and cascading chevron amplification. The success of the current experiments in achieving relatively high logic gate effectiveness has paved the path to the future dawn of thermal computing.

## Supplementary information

Supplementary Video 1.

Supplementary Video 2

Supplementary Video 3

Supplementary Video 4

Supplementary Video 5

Supplementary Video 6
